# Factors associated with achieving intraocular pressure lower than 15 mmHg by Trabectome surgery in primary open-angle glaucoma

**DOI:** 10.1038/s41598-021-93711-w

**Published:** 2021-07-12

**Authors:** Kentaro Nakamura, Rio Honda, Shoichi Soeda, Norihiro Nagai, Osamu Takahashi, Kazuaki Kadonosono, Yoko Ozawa

**Affiliations:** 1 Department of Ophthalmology, St. Luke’s International University and Hospital, 9-1 Akashi-cho, Chuo-ku, Tokyo, 104-8560 Japan; 2 Department of Ophthalmology and Micro-Technology, Yokohama City University Medical Center, 4-57 Urafune-cho, Minami-ku, Yokohama, Kanagawa 232-0024 Japan; 3 Laboratory of Retinal Cell Biology, Department of Ophthalmology, Keio University School of Medicine, 35 Shinanomachi, Shinjukuku, Tokyo 160-8582 Japan; 4 Graduate School of Public Health, St. Luke’s International University, 9-1 Akashi-cho, Chuo-ku, Tokyo, 104-8560 Japan; 5 Laboratory of Retinal Cell Biology, St. Luke’s International University, 9-1 Akashi-cho, Chuo-ku, Tokyo, 104-8560 Japan

**Keywords:** Diseases, Medical research

## Abstract

To assess good prognostic factors of Trabectome surgery in primary open-angle glaucoma (POAG), clinical records of patients with POAG who underwent Trabectome surgery with/without cataract surgery as the first additive therapy to eye drops between January 2015 and March 2018 were retrospectively reviewed. Overall, data of 79 eyes (79 patients; 50 men; mean age, 68.0 years) up to postoperative 24 months were analyzed. Their mean intraocular pressure (IOP) was 20.4 ± 6.0 mmHg at baseline. Forty-two eyes (53.2%) achieved an IOP < 15 mmHg and ≥ 20% reduction from baseline without additional treatments. Phakic eyes had a better survival probability than pseudophakic eyes after adjusting for age, sex, baseline IOP, best-corrected visual acuity, and eye drop score (hazard ratio 3.096; 95% confidence interval [95% CI] 1.367–7.013; P = 0.007). Phakic eyes treated with combined Trabectome and cataract surgeries (mean survival time, 22.250 months; 95% CI 17.606–26.894) had a better survival probability than pseudophakic eyes treated with Trabectome surgery only (mean survival time, 12.111 months; 95% CI 8.716–15.506; P = 0.009) after the adjustment. Among the eyes treated with Trabectome surgery only, phakic eyes required significantly less additional treatments than pseudophakic eyes (P = 0.04). Trabectome surgery may be indicated for phakic eyes with POAG in addition to eye-drop therapy.

## Introduction

Glaucoma is a blinding disease due to optic nerve degeneration^1,2^; Tham et al. reported the global prevalence of all types of glaucoma at 3.54% and that of primary open-angle glaucoma (POAG) at 3.05% for people aged 40–80 years^3^. The only generally accepted treatment is lowering the intraocular pressure (IOP), and the first-line treatment worldwide is daily eye-drop application^4,5^. However, low adherence to continuous eye-drop treatment and/or IOP fluctuations between eye-drop applications may cause glaucoma progression, leading to increased visual-field defect. Therefore, alternative treatments, such as incisional glaucoma surgery, have also been indicated for patients who fail to achieve an acceptably low IOP.

IOP is determined by the balance of production and outflow of the aqueous humor, and most surgeries are performed to increase the outflow. In contrast to the traditional trabeculectomy and implantation of glaucoma drainage device implants, micro- or minimally invasive glaucoma surgeries (MIGSs) require no manipulation of the sclera and conjunctiva^6–9^; thus, patients are treated more safely and recover more rapidly, although the IOP lowering effect may be less pronounced than that achieved with the abovementioned surgeries^6^. One type of MIGS, Trabectome surgery^6,10,11^, was approved by the US Food and Drug Administration in 2004^6^; the device consists of an electrode to ablate the trabecular meshwork, not only to make an incision, and continuous infusion and aspiration to remove the debris, both performed from the anterior chamber side, ab interno. Trabectome surgery has been found to reduce nocturnal IOP peaks and diurnal IOP fluctuations, and stabilize the IOP^12^. Thus, Trabectome surgery could overcome the limitations of eye-drop treatments and could be one of the most attractive options for patients who cannot be sufficiently treated solely by using eye drops.

Considering that the IOP lowering effect may be lower in Trabectome surgery^6^, and that Trabectome surgery has been reported to be effective for eyes with relatively lower IOPs^12^, it may be applied to patients whose preoperative IOP is not extremely high; therefore, whether the IOP becomes lower than 21 mmHg using this treatment may not be a suitable assessment criterion for determining the effects of Trabectome surgery. In addition, a recent population-based study showed that Japanese people have a lower average IOP (14.5 mmHg) than Western people^13,14^. Normal-tension glaucoma, categorized as POAG, is associated with an IOP lower than 21 mmHg^15^, in which the target IOP of the treatment would be set lower e.g., at 9–12 mmHg^16^. The survival probability may be better determined by setting the assessment criterion at a lower IOP, such as at < 15 mmHg. Moreover, whether it has long-lasting effects would also be important for glaucoma surgeries.

In this study, we retrospectively evaluated patients with POAG who were treated with Trabectome surgery as the first option when eye-drop therapy did not suffice. The predictive factors for patients whose IOP was successfully lowered, particularly, to levels lower than 15 mmHg with no additional treatments including additional IOP-lowering medications and any ocular surgeries could help determine the application of surgery and secure patient informed consent before surgery in the daily clinic.

## Results

A total of 107 eyes of 79 patients underwent Trabectome surgery for POAG. Among them, 28 patients underwent bilateral surgery; for these patients, only the right eyes were included. Thus, the data of 79 eyes of 79 patients (50 men, 63.3%; mean age 68.0 ± 11.5 [range 40–92; median 69] years) were analyzed (Table 1). The mean best-corrected visual acuity (BCVA) was 0.17 ± 0.45 (range − 0.18 to 2.00; median 0.00) in LogMAR. The mean IOP was 20.4 ± 6.0 (range 11–41; median 20.0) mmHg. The mean eye-drop score was 3.7 ± 0.8 (range 2–6; median 4), and there were 44 phakic eyes (55.72%) at baseline (Table 1). Prostaglandin-related ophthalmic solutions (PGs), which are the first-line eye-drop choice for glaucoma worldwide including Japan^4,5^, were used in all eyes preoperatively. Other preoperative eye drops included β adrenergic antagonists (β blockers), carbonic anhydrase inhibitors (CAI), α2 selective adrenergic agonists (α2 stimulants), and Rho kinase inhibitors (ROCK inhibitor). In addition, oral CAI was prescribed in nine patients transiently before the surgery; oral CAI use was not included in the eye-drop score. For combination eye drops, the eye-drop score was counted as two. No eyes had been previously treated with IOP lowering surgeries including laser therapy. The mean follow-up duration was 17.4 ± 9.7 (range 0.25–24; median 24) months. Twenty patients dropped out before the study endpoint at 24 months after the surgery.

**Table 1:** Baseline characteristics.

Age (years, [median])	68.0 ± 11.5 (40 to 92) [69]
Sex (men, eyes [%])	50 (63.3)
BCVA (LogMAR, [median])	0.17 ± 0.45 (− 0.18 to 2.00) [0.00]
IOP (mmHg, [median])	20.4 ± 6.0 (11 to 41) [20.0]
Eye drop score [median]	3.7 ± 0.8 (2 to 6) [4]
Phakic eyes (eyes [%])	45 (57.0)
**Medications**	
PGs (eyes [%])	79 (100.0)
β blockers (eyes [%])	64 (81.0)
CAI (eyes [%])	70 (88.6)
α2 stimulant (eyes [%])	43 (54.4)
ROCK inhibitor (eyes [%])	21 (26.6)
Non-selective muscarinic receptor stimulant (eyes [%])	1 (1.3)
Oral CAI (eyes [%])	9 (11.4)
Follow-up duration (months, [median])	17.4 ± 9.8 (0.25 to 24) [24]
Drop-out before month 24 (eyes [%])	20 (25.3)

Dara are shown in mean ± standard deviation (SD) and median.

*BCVA* best-corrected visual acuity, *IOP* intraocular pressure, *PGs* prostaglandin-related ophthalmic solutions, *β blockers* β adrenergic antagonists, *CAI* carbonic anhydrase inhibitors, *α2 stimulant* α2 selective adrenergic agonists, *ROCK* inhibitor Rho kinase inhibitor.

In all the patients, the mean IOPs decreased to 13.5 ± 3.6 (median 13) mmHg and 12.8 ± 2.0 (median 12) mmHg, with or without postoperative additional medications and/or any ocular surgery (both P < 0.01). The mean BCVAs were 0.18 ± 0.50 (median 0.00) and 0.20 ± 0.56 (median 0.00), at 12 and 24 postoperattive months, respectively (data not shown). There were no intraoperative complications. Mild hyphema was observed in 26 eyes (32.9%), including one eye with slight vitreous hemorrhage, which disappeared in 17 eyes by one postoperative week.

**Figure 1:**
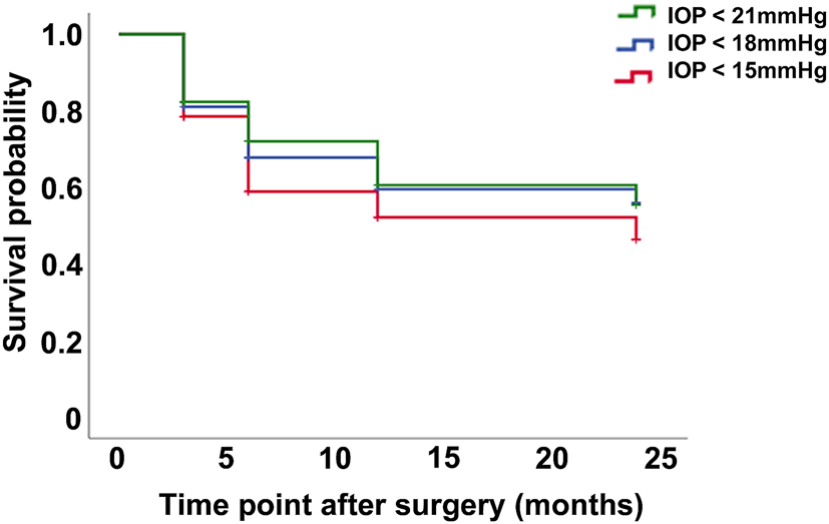
Kaplan–Meier survival analysis after the Trabectome surgery. Survival was defined by a postoperative intraocular pressure (IOP) < 21 mmHg and ≥ 20% reduction (green), IOP < 18 mmHg and ≥ 20% reduction (blue), and IOP < 15 mmHg and ≥ 20% reduction (red), all compared with baseline values with no additional treatments including IOP-lowering eye drops, oral CAI, or any ocular surgeries or laser therapies.

The Kaplan–Meier survival analysis of the 79 eyes showed that 48 (60.8%) eyes survived at 24 months (mean survival time, 17.053 months; 95% confidence interval [95% CI] 14.956–19.150) when survival was defined as a postoperative IOP < 21 mmHg and reduction in IOP ≥ 20% compared with the baseline values; 48 eyes (60.8%) survived (mean survival time, 16.635 months; 95% CI 14.479–18.791) with the definition of an IOP < 18 mmHg and reduction in IOP ≥ 20%; and 42 eyes (53.2%) survived (mean survival time, 15.606 months; 95% CI 13.293–17.918) with the definition of an IOP < 15 mmHg and reduction in IOP ≥ 20%, all with no additional treatments including IOP-lowering eye drops, oral CAI, or any ocular surgeries or laser therapies (Fig. 1).

According to the IOP definition, the number of failed eyes was 8 (10.1%) with an IOP ≥ 21 mmHg, 12 (15.2%) with an IOP ≥ 18 mmHg, 23 (29.1%) with an IOP ≥ 15 mmHg, and 24 (30.4%) with a reduction in IOP  < 20%. Additional eye drops were prescribed for 11 eyes (13.9%), and additional ocular surgeries were performed in 8 eyes (10.1%), both during the 24-month period after the Trabectome surgery. Overall, 17 eyes (21.5%) needed additional treatments during these 24 months and were also deemed as failures.

The baseline characteristics of the eyes that were related to survival or failure with an IOP < 15 mmHg and reduction in IOP ≥ 20% compared with the baseline values, with no additional treatments including IOP lowering medications and/or any ocular surgeries were analyzed using the Cox proportional hazards model (Table 2). Psedophakic eye was significantly related to worse survival probability (hazard ratio 3.096; 95% CI 1.367–7.013; P = 0.007).

**Table 2:** Factors associated with achieving IOP < 15 mmHg and ≥ 20% reduction with no additional treatments compared with baseline.

	Hazard ratio	95% confidence interval	P value
Age	0.982	0.949–1.017	0.312
Sex (men)	0.950	0.463–1.948	0.888
BCVA	1.627	0.847–3.125	0.144
IOP	0.943	0.877–1.014	0.115
Eye drop score	0.964	0.627–1.483	0.868
Pseudophakic eye	3.096	1.367–7.013	0.007*

Cox proportional hazards model.

*BCVA* best-corrected visual acuity, *IOP* intraocular pressure.

**P < 0.01.

The course of the mean IOP in the eyes that survived and failed with an IOP < 15 mmHg and reduction in IOP ≥ 20% with no additional treatments was analyzed (Supplementary Fig. 1). The eyes that survived had a significantly lower IOP at one postoperative month (mean ± SE [median] IOP of survival vs failure; 12.8 ± 0.68 [12] mmHg and vs 13.5 ± 0.48 [13.5] mmHg; P = 0.039), although the mean baseline IOP was similar (20.7 ± 0.81 [20] mmHg and vs 20.0 ± 1.09 [20.5] mmHg; P = 0.526). Moreover, mean IOP reductions compared with baseline was greater in the eyes that survived as early as one week after the Trabecome surgery, and the condition continued to one month (Supplementary Table 1).

**Figure 2:**
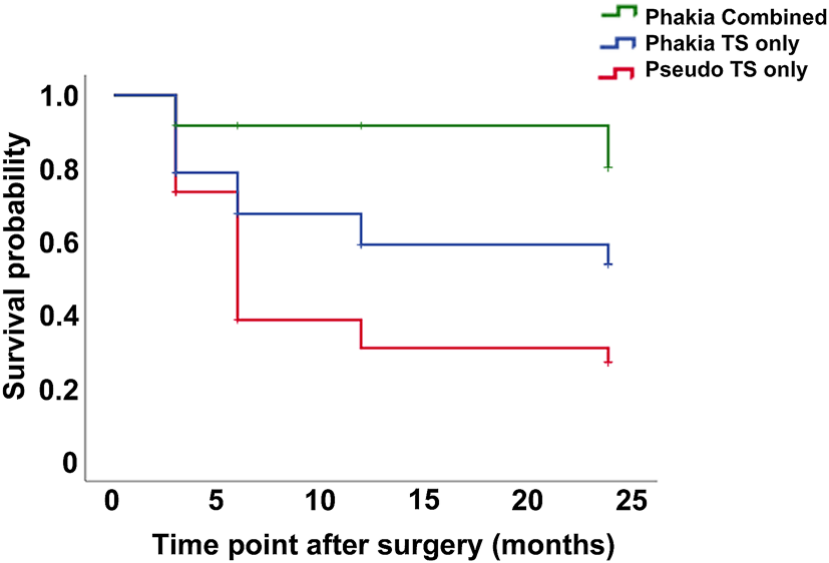
Kaplan–Meier survival analysis according to the preoperative lens status and surgical procedures, with survival defined as intraocular pressure (IOP) < 15 mmHg and ≥ 20% compared reduction with baseline and with no additional treatments. Data on phakic eyes that underwent Trabectome surgery (TS) and combined cataract surgery (green), phakic eyes that underwent TS only (blue), and pseudophakic eyes that underwent TS only (red) are shown.

Kaplan–Meier survival analysis defined by an IOP < 15 mmHg and a reduction in IOP ≥ 20% with no additional treatments including IOP lowering medications and/or any ocular surgeries was performed according to whether the eyes were phakic or pseudophakic, and among the phakic eyes, whether the patients underwent combined Trabectome and cataract surgeries or only Trabectome surgery (Fig. 2). Among the 12 phakic eyes treated with combined surgery, 10 eyes (83.3%) survived at 24 months (mean survival time, 22.250 months; 95% CI 17.606–26.894). In the other 33 phakic eyes treated with Trabectome surgery only, 19 eyes (57.6%) survived (mean survival time, 17.182; 95% CI 13.768–20.596), and in the 34 pseudophakic eyes treated with Trabectome surgery only, 12 eyes (35.3%) survived (mean survival time, 12.111 months; 95% CI 8.716–15.506) at 24 months.

The Cox proportional hazards model showed that pseudophakic eyes treated with Trabectome surgery only had a significantly worse prognosis than phakic eyes treated with combined Trabectome and cataract surgeries (hazard ratio, 0.133; 95% CI 0.029–0.612; P = 0.009) (Table 3). Also, there was a trend of worse prognosis in the pseudophakic eyes when compared with phakic eyes treated with Trabectome surgery only (hazard ratio 0.461; 95% CI 0.189–1.122; P = 0.088).

**Table 3:** Comparisons between the surgical procesures for achieving IOP < 15 mmHg and ≥ 20% reduction with no additional treatments.

	Hazard ratio	95% confidence interval	P value
TS only in pseudophakic eyes	1.000		
TS only in phakic eyes	0.461	0.189–1.122	0.088
Combined surgery in phakic eyes	0.133	0.029–0.607	0.009*

Cox proportional hazards model adjusted for age, sex, best-corrected visual acuity, intraocular pressure, and eye drop score at baseline. *P < 0.05.

Interestingly, no phakic eyes treated with combined Trabectome and cataract surgeries needed additional treatments. Moreover, among the eyes that underwent only Trabectome surgery, additional treatments with eye drops and/or surgeries were needed in 3/33 phakic eyes (9.1%) and 14/34 pseudophakic eyes (38.2%) during follow-up; there was a significant difference in the requirement for additional treatments (P = 0.004) (Table 4). Overall, additional treatments were required in 17 eyes (21.5%).

**Table 4:** Requirement of additional treatments in the eyes that underwent Trabectome surgery alone in phakic and pseudophakic eyes.

	Phakia	Pseudophakia	P
Eyes (%)	33 (49.3)	34 (50.7)	–
Overall (eyes [%])	3 (9.1)	14 (38.2)	0.004**
Eye drops (eyes [%])	2 (6.1)	9 (26.5)	0.04*
Surgeries (eyes [%])	2 (6.1)	6 (17.6)	0.26

Eyes which required both eye drops and surgeries were included in both categories. Fisher's exact test. *P < 0.05.

## Discussion

Trabectome surgery, used as first-line treatment for IOP lowering, successfully reduced the IOP in patients with POAG at 24 months. In particular, 61% of the patients retained IOP < 18 mmHg, and 53% retained IOP < 15 mmHg, both of which also met the definition of IOP reduction ≥ 20% compared with baseline with no additional treatments during 24 months of follow-up. The eyes that survived with the latter definition showed a significantly greater reduction in IOP as early as one postoperative week, compared with baseline. The phakic eyes at baseline had a significantly better survival probability compared with phakic eyes. In particular, the phakic eyes that underwent combined cataract surgery more frequently survived at 24 months with a definition of IOP < 15 mmHg, ≥ 20% reduction from baseline, and no additional treatments including IOP lowering medications and/or any ocular surgeries, compared with pseudophakic eyes that underwent Trabectome surgery. Overall, 21.5% of the included eyes required additional treatments, including additional eye drops and surgeries; however, among the eyes that underwent Trabectome surgery only, phakic eyes needed additional treatments less frequently (9.1%) than pseudophakic eyes (38.2%).

The survival probability after Trabectome surgery in patients with a postoperative IOP < 21 mmHg and ≥ 20% reduction from the preoperative IOP at 24 months was 61%, comparable to that previously reported by Kono et al.^17^. The probability with the same definition by year 2 reported by other groups was approximately 70% and appeared to be better than the current result^18–20^. However, this could be because the reports involved both POAG and secondary open angle glaucoma (SOAG), the latter of which has a better prognosis after Trabectome surgery^21,22^.

The mean preoperative IOP included in the current study was 20.4 mmHg, and it was relatively lower than those reported at the introduction of the Trabectome surgery, which mainly included eyes with a preoperative IOP of approximately 25 mmHg^11,23^. The application of surgery may be expanded to milder cases in response to dissemination of the surgical technique, and the definition of survival may better be set sufficiently low considering the clinical impact.

Thus, we analyzed the data with more strict definitions than the cutoff value of 21 mmHg. The survival probability defined as a postoperative IOP < 18 mmHg and ≥ 20% reduction from the preoperative IOP in the current study was 61% and better than that previously reported by Ahuja et al., which was 22%^24^. The same definition was applied to studies including both POAG and SOAG, and reported to be 51% by Kono et al.^17^ and 40% by Kinoshita-Nakano et al.^25^. Therefore, the success probability of 53% in the current study with a postoperative IOP  <  15 mmHg and ≥ 20% reduction from the preoperative IOP among patients with only POAG was equal to or greater than those previously reported.

Phakic eyes had a better prognosis both in terms of survival probability and no requirement of additional treatments, partly because phakic eyes involved those treated with combined cataract surgery, which itself can reduce the IOP^26^. In fact, it has been reported that combined surgery was the key factor for a better survival probability in phakic eyes in a previous long-term analysis^17^. However, the data were those of patients with either POAG or SOAG, such as developmental glaucoma, and not of patients with POAG only. In addition, combined Trabectome and cataract surgeries achieved only less than 1 mmHg reduction in the IOP compared with Trabectome surgery alone as previously reported^27^. Importantly, the requirement of additional treatments was not observed in the phakic eyes treated with combined Trabectome and cataract surgery, and significantly less frequent in phakic than in pseudophakic eyes that underwent Trabecrome surgery only. Clinicians may hesitate to perform open surgery in phakic eyes; however, Trabectome surgery may be more suitable for phakic eyes than pseudophakic eyes, not necessarily combined with cataract surgery, although further studies are warranted.

In Trabectome surgery, the trabecular meshwork is dissected, and the tissue is ablated; therefore, it differs from trabeculotomy both ab interno and ab externo. It could be rather similar to trabeculectomy ab externo; however, the latter aims to create an outflow of the aqueous humor into the bleb through the scleral flap and differs from Trabectome surgery, which utilizes Schlemm’s canal for outflow^21^. Given that aqueous-humor outflow is negatively correlated with postoperative IOP and positively correlated with flow speed of the episcleral veins^28^, a lower flow resistance of Schlemm’s canal or following veins would be of importance for lowering the IOP after Trabectome surgery. Pseudophakic eyes might have a greater flow resistance of Schlemm’s canal and/or following veins because of postoperative mild but inevitable inflammation caused by the previous cataract surgery; thus, the prognosis is limited, although the inflammation would be minimal, given the current progress in surgical technology of cataract extraction. Future studies are required to clarify this.

The limitations of the current study included: the relatively small sample size, particularly for patients who underwent combined cataract surgery; retrospective design, performance of all procedures by a single surgeon in a single center, and determination of additional treatments during the follow-up by both the IOP and surgeon’s judgement. However, because the same surgeon examined all the patients, the bias of the difference in the clinical determination may have been minimal.

In conclusion, Trabectome surgery was performed in eyes with POAG whose preoperative IOP was approximately 20 mmHg. The survival probability with no additional treatment at 24 months was 53% when defined by a postoperative IOP < 15 mmHg and ≥ 20% reduction from the preoperative IOP. The probability of meeting the criteria was greater in the phakic eyes. In particular, the probability was significantly higher in the phakic eyes treated with combined cataract surgery than the pseudophakic eyes. Additional treatments were not required in the phakic eyes that were treated with combined surgery and less frequently required for the phakic eyes than pseudophakic eyes that only underwent Trabectome surgery. Thus, Trabectome surgery even without cataract extraction may be indicated for phakic eyes whose POAG cannot be sufficiently controlled by solely using eye drops.

## Methods

The procedures of this retrospective study adhered to the tenets of the Declaration of Helsinki, and approval to perform this study was obtained from the St. Luke’s International University Ethics Committee (approval number: 20-R048).

### Patients

The analysis was based on a detailed medical chart review. The study included patients with POAG diagnosed using Anderson and Patella’s criteria in perimetry who underwent Trabectome surgery combined with or without cataract surgery (as the first additional treatment to eye drops for lowering the IOP) between January 2015 and March 2018 in the Department of Ophthalmology, St. Luke’s International Hospital (Tokyo, Japan). If patients underwent Trabectome surgery in both eyes, only the right eye data were included in the analyses. Patients who had no history of ocular surgery within 1 month prior to the Trabectome surgery were included. Patients who were lost to follow-up before 24 months mostly because of reverse referral to the local clinics were included. Patients who had secondary or etiology-unknown glaucoma were excluded.

### Eye examinations

All patients underwent complete ophthalmologic examinations, including BCVA measurement with a refraction test, IOP measurements using Goldmann’s applanation tonometer, slit-lamp examinations, and indirect ophthalmoscopy after pupil dilation with 0.5% tropicamide.

### Surgery

In all patients, Trabectome surgery was performed by an experienced surgeon (SS) using the Trabectome system (Neomedix Inc., Tustin, CA, USA) under Hill surgical gonioprism (Ocular Instruments, Bellevue, WA, USA), combined or not with cataract surgery. The trabecular meshwork was ablated in the range of 120° with a fixed power of 0.8 W. In the eyes treated with combined cataract surgery, Trabectome surgery was performed prior to phacoemulsification.

### Postoperative treatments

All patients continued using the same preoperative glaucoma eye drops after the surgery, following the approved dosage and use for individual eye drops. Moreover, 0.1% betamethasone and nepafenac to resolve surgery-related inflammation and 0.3% gatifloxacin hydrate to avoid infection, as well as 1% pilocarpine, were used topically four times a day for 1 month. The need for additional treatments, including IOP-lowering eye drops and oral CAI, and glaucoma or other ocular surgeries or laser therapies were determined by the surgeon who performed the surgery and follow up (SS).

### Definition of treatment success (survival)

Survival was defined as a postoperative IOP < 21 mmHg and ≥ 20% reduction, or IOP < 18 mmHg and ≥ 20% reduction, or IOP < 15 mmHg and ≥ 20% reduction, all compared with the baseline values, with no need for additional treatments including IOP-lowering eye drops, oral CAI, and glaucoma and other ocular surgeries or laser therapies. Eyes that needed these additional medications and/or surgeries were considered failed eyes. There were no eyes that progressed to no light perception. Survival or failure was determined at 3, 6, 12, and 24 months after Trabectome surgery.

### Statistical analyses

Data are expressed as mean ± standard deviation (SD) or mean ± standard error (SE) or median. Analyses via the Wilcoxon signed rank test, Kaplan–Meier survival analysis, cox proportional hazard model, Mann–Whitney U test, and Fisher’s exact test were performed using SPSS (version 25.0, SPSS Japan, Tokyo, Japan). A P value < 0.05 was considered statistically significant.

## Supplementary Information

Supplementary Information 1.

## Data Availability

The datasets generated or analyzed during the current study are available from the corresponding author on reasonable request.
