# Supplementary material for: Factors associated with achieving intraocular pressure lower than 15 mmHg by Trabectome surgery in primary open-angle glaucoma
Source: Sci Rep. 2021 Jul 12;11:14308. doi: 10.1038/s41598-021-93711-w (PMC8275622; doi:10.1038/s41598-021-93711-w)
Supplement: Supplementary file 1 — Supplementary Information 1. [file 41598_2021_93711_MOESM1_ESM.pdf]

Factors associated with achieving intraocular pressure lower than 15 mmHg by Trabectome surgery in primary open-angle glaucoma

Kentaro Nakamura[1,2], Rio Honda[1], Shoichi Soeda[1], Norihiro Nagai[3], Osamu Takahashi[4], Kazuaki Kadonosono[2], Yoko Ozawa[1,3,5*],

[1]Department of Ophthalmology, St. Luke's International Hospital, 9-1 Akashi-cho, Chuo-ku, Tokyo 104-8560, Japan.
[2]Department of Ophthalmology and Micro-technology, Yokohama City University Medical Center, 4-57 Urafune-cho, Minami-ku, Yokohama city, Kanagawa 232-0024, Japan.
[3]Department of Ophthalmology, Keio University School of Medicine, 35 Shinanomachi, Shinjukuku, Tokyo 160-8582, Japan.
[4]Graduate School of Public Health, St. Luke's International University, 9-1 Akashi-cho, Chuo-ku, Tokyo 104-8560, Japan.
[5]St. Luke's International University, 9-1 Akashi-cho, Chuo-ku, Tokyo 104-8560, Japan.

***Corresponding author:**
Yoko Ozawa, M.D., Ph.D.
Professor and Director
Department of Ophthalmology,
St. Luke's International University and Hospital
E-mail: ozaway@luke.ac.jp
Lab Chief & Associate Professor
Laboratory of Retinal Cell Biology,
Department of Ophthalmology,
Keio University School of Medicine
E-mail: ozawa@a5.keio.jp
ORCID: 0000-0003-4797-5705

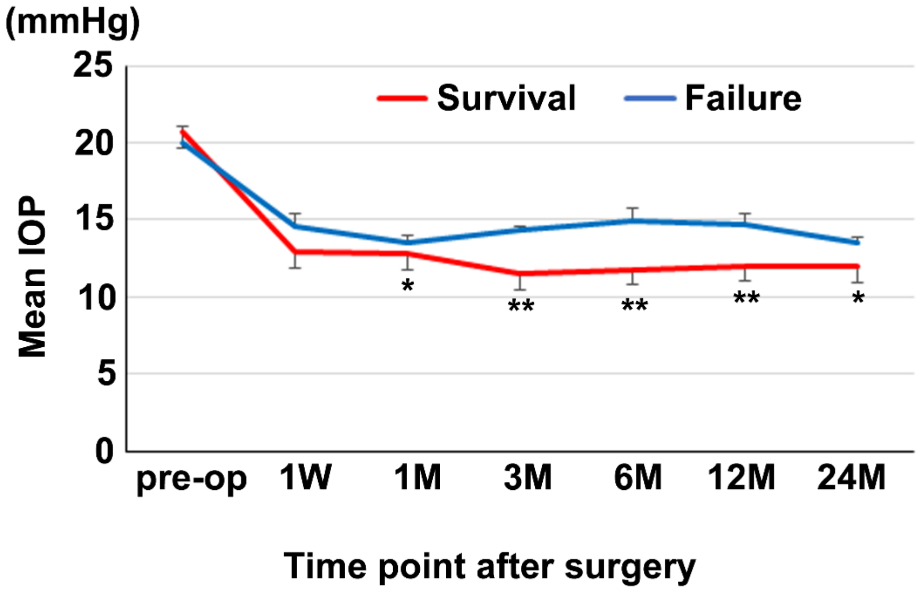

Supplementary Figure 1. The course of mean intraocular pressure (IOP) after surgery

Data are expressed as mean ± standard error (SE). Mean IOPs after surgery were shown in the eyes that survived (red) and failed (blue) defined by the criteria of IOP < 15 mmHg and ≥ 20 % reduction compared with baseline with no additional treatments at 24 months. There were significant differences between the survived and failed group at 1, 3, 6, 12, and 24 months after surgery. Mann-Whitney test between groups. *P < 0.05, **P < 0.01.

Supplementary Table 1. Mean postoperative reductions in intraocular pressure at each time point after surgery

	Survived	Failed	P
at 1 week (mmHg)	7.8 ± 0.96 [7]	5.4 ± 0.91 [5]	0.047*
at 1 month (mmHg)	9.1 ± 0.92 [8]	6.5 ± 0.88 [5]	0.045*

Dara are shown in mean ± standard error (SE) and median. Survival was defined by the criteria of IOP < 15 mmHg and ≥ 20 % reduction compared with baseline with no additional treatments at 24 months. Reductions in intraocular pressure was calculated comparing before and after the surgery at each time point. Mann Whitney U test. *P<0.05.
